# Advancements in Perovskite Nanocrystal Stability Enhancement: A Comprehensive Review

**DOI:** 10.3390/nano13111707

**Published:** 2023-05-23

**Authors:** Xuewen Liu, Eun-Cheol Lee

**Affiliations:** 1 Department of Nano Science and Technology, Graduate School, Gachon University, Seongnam-si 13120, Republic of Korea; 2 Department of Physics, Gachon University, Seongnam-si 13120, Republic of Korea

**Keywords:** perovskite nanocrystals, stability, enhancement

## Abstract

Over the past decade, perovskite technology has been increasingly applied in solar cells, nanocrystals, and light-emitting diodes (LEDs). Perovskite nanocrystals (PNCs) have attracted significant interest in the field of optoelectronics owing to their exceptional optoelectronic properties. Compared with other common nanocrystal materials, perovskite nanomaterials have many advantages, such as high absorption coefficients and tunable bandgaps. Owing to their rapid development in efficiency and huge potential, perovskite materials are considered the future of photovoltaics. Among different types of PNCs, CsPbBr_3_ perovskites exhibit several advantages. CsPbBr_3_ nanocrystals offer a combination of enhanced stability, high photoluminescence quantum yield, narrow emission bandwidth, tunable bandgap, and ease of synthesis, which distinguish them from other PNCs, and make them suitable for various applications in optoelectronics and photonics. However, PNCs also have some shortcomings: they are highly susceptible to degradation caused by environmental factors, such as moisture, oxygen, and light, which limits their long-term performance and hinders their practical applications. Recently, researchers have focused on improving the stability of PNCs, starting with the synthesis of nanocrystals and optimizing (i) the external encapsulation of crystals, (ii) ligands used for the separation and purification of nanocrystals, and (iii) initial synthesis methods or material doping. In this review, we discuss in detail the factors leading to instability in PNCs, introduce stability enhancement methods for mainly inorganic PNCs mentioned above, and provide a summary of these approaches.

## 1. Introduction

**Figure 1:**
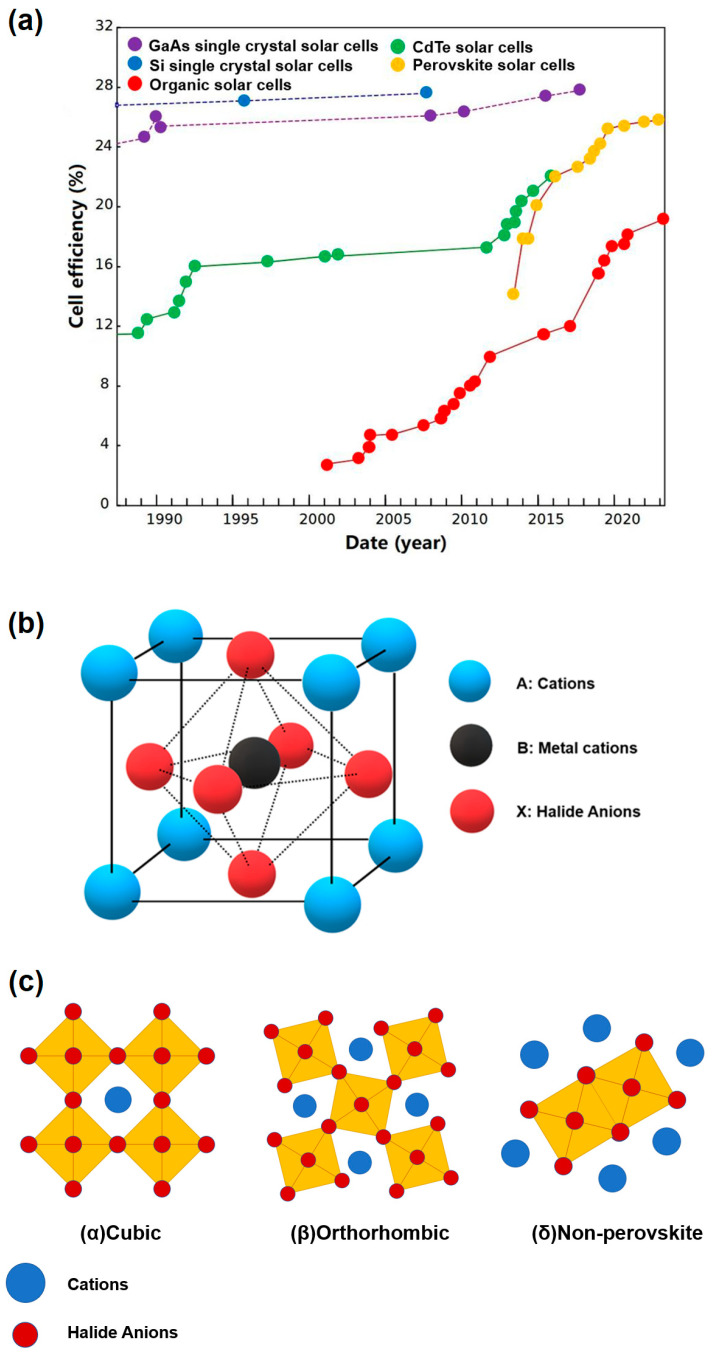
(**a**) Chart of highest confirmed conversion efficiencies for a range of photovoltaic technologies across decades. (**b**) Crystal structure of perovskite. (**c**) Schematic diagram of perovskite phase.

The term “perovskite” originally referred to a mineral known as CaTiO_3_. In 1839, German mineralogist Gustav Rose discovered perovskites in the Ural Mountains of Russia. In honor of the Russian mineralogist Lev Perovski (1792–1856), the material was named “perovskite”. Later, materials with a crystal structure similar to CaTiO_3_ were classified as “perovskite” in mineralogy, with the chemical formula of ABX_3_, as shown in Figure 1 [1,2,3]. The success of metal halide perovskites in photovoltaic devices has led to increased interest in this type of material among scientists in various research fields, making it one of the most studied research topics worldwide in recent years [4,5,6]. In the past decade, perovskites have been composed of monovalent organic or inorganic cations (such as methylammonium (MA), formamidinium (FA), Cs^+^, and Rb^+^) at the A site, divalent metal cations (such as Ge^2+^, Cu^2+^, Pb^2+^, and Sn^2+^) at the B site, and halide anions (Cl^−^, Br^−^, and I^−^) at the X site.

In recent years, perovskites have gained widespread utilization in the materials industry. Perovskite materials exhibit immense potential across various fields, extending beyond the photovoltaic sector. They find applications in photocatalysis, photodetection, and light-emitting diodes (LEDs). Notably, in the realm of photovoltaics, perovskite solar cells have witnessed a remarkable increase in power conversion efficiency, rising from 3.8% to 25.73% [7,8,9,10,11,12,13,14,15,16,17,18].

Perovskite research expanded to the nanoscale with the introduction of Perovskite nanocrystals (PNCs) in 2014 [19]. PNCs possess the optoelectronic properties of bulk perovskite materials and the quantum confinement effects of materials at the nanoscale, resulting in tunable bandgaps, narrow emissions, and strong light-absorption coefficients. Unlike traditional quantum dots, such as sulfide and selenide quantum dots, PNCs have high defect tolerance [20,21,22]. The dimensions and shapes of PNCs can be easily adjusted to 0D quantum dots, 1D nanowires [23,24,25] and nanorods [26,27,28], 2D nanosheets [29,30,31,32] and nanoplatelets [33,34], and 3D nanocubes [35,36] by modifying the reaction conditions. Recently, the easy synthesis of PNCs with various dimensions and shapes has attracted considerable attention [37,38]. In this section, we discuss wet synthesis techniques, such as hot injection [39,40], ligand-assisted reprecipitation (LARP) [41,42], emulsion processes [43,44], ultrasound- [45,46] and microwave-based methods [47,48], solvothermal approaches [49,50,51], microfluidic techniques [52,53,54], and template-assisted methods [55,56,57]. Each of these methods has its own set of advantages and disadvantages. To summarize, for instance, the hot injection method can yield high-quality nanoparticles, but it entails a complex and costly process. LARP is cost-effective and reproducible, but it is limited in terms of nanoparticle shape. Emulsion processes offer versatility in nanoparticle size and shape, but removing the surfactants can be challenging. Both ultrasound and microwave methods are rapid but present difficulties in controlling nanoparticle shape. Solvothermal approaches can yield high-purity products, but they come with high costs and safety concerns. Microfluidic techniques provide control over nanoparticle size and morphology, but the design and fabrication process is complex. Template-assisted methods also offer high product yields, but removing the template presents another challenge [58,59,60,61,62]. Researchers must weigh these factors and make tradeoffs when selecting methods to synthesize products with desired morphologies of the highest quality.

CsPbBr_3_ nanocrystals, a specific class of all-inorganic PNCs, exhibit several advantages over other PNCs, which make them highly attractive for various applications. CsPbBr_3_ nanocrystals exhibit a high photoluminescence quantum yield (PLQY), making them exceptionally efficient at converting absorbed light into emitted light. Furthermore, they have a great capacity for light absorption within the perovskite film. This property is particularly useful for optoelectronic applications, such as LEDs and solar cells. The emission spectrum of CsPbBr_3_ nanocrystals is characterized by a narrow bandwidth, resulting in highly pure and saturated colors [63,64]. This is particularly beneficial for applications requiring color purity, such as display technologies and solid-state lighting. CsPbBr_3_ nanocrystals possess an easily tunable bandgap, which allows for the adjustment of their optical properties by altering their composition or size. This flexibility makes them suitable for a wide range of applications with different spectral requirements, such as photodetectors and lasers [23,65,66,67]. CsPbBr_3_ nanocrystals can be synthesized using simple and low-cost methods, such as hot injection and solvothermal techniques, making them an attractive option for scalable production [63,64,68]. CsPbBr_3_ nanocrystals offer a combination of enhanced stability, high PLQY, narrow emission bandwidth, tunable bandgap, and ease of synthesis, which distinguishes them from other PNCs and makes them suitable for various applications in optoelectronics and photonics.

Although perovskite materials have seen rapid development and enormous potential in areas, such as solar cells and LEDs [69], these materials continue to face significant challenges, particularly in the realm of large-area devices, with long-term stability and thermal stability being critical issues [70,71,72]. This review discusses the existing stability problems and influencing factors in perovskite nanomaterials, especially inorganic PNCs (CsPbBr_3_); furthermore, it presents the methods followed by researchers to address the low stability of perovskite nanomaterials in recent years, as well as the prospects for the future of perovskite nanomaterials.

## 2. Factors Influencing the Stability of PNCs

Stability is an important issue that affects the performance of perovskite nanomaterials and their component devices, as well as their future commercialization. This section reviews the degradation mechanisms of perovskites and the reasons for their degradation, including the crystal structure stability, environmental factors, and operational thermal stability.

### 2.1. Structure Instability

The instability of PNCs is mainly attributed to the intrinsic instability of the perovskite crystal structure, presence of defects and impurities, stability arising from the crystal–ligand interface, and interactions with the environment. The ideal skeleton structure of perovskites consists of a central cation coordinated with six halide ions located around it, forming a BX_6_ octahedral configuration. However, in practice, the common skeletal structure of calcium titanium oxide is a distorted octahedron that forms an orthorhombic phase [73,74,75]. It is difficult to achieve a highly symmetrical octahedral structure, and this distortion can cause the optical or electronic properties to deviate from the original target by varying degrees [75,76]. To evaluate the stability of the calcium titanium oxide structure, a tolerance factor is typically used, expressed as follows [1]:

(1) $$t=\frac{r_{A}+r_{X}}{\sqrt{2}(r_{B}+r_{X})},$$

where *r*_A_, *r*_B_, and *r*_X_ represent the radii of the elements A, B, and X, respectively. For an ideal regular octahedral crystal skeleton, *t* = 1. Previous studies have shown that calcium titanium oxide skeleton structures with 0.9 ≤ *t* ≤ 1.0 are relatively ideal, and those with 0.71 ≤ *t* ≤ 0.9 indicate a slightly distorted orthorhombic structure. If *t* is outside this range, the calcium titanium oxide skeleton is not octahedral, which can lead to poor optical and electronic properties of the material [1].

The perovskite crystal structure is composed of a metal halide octahedral framework, which is susceptible to distortion and degradation under various conditions. The instability of the crystal structure is aggravated by the presence of defects and impurities, such as vacancies, interstitials, and dopants, which can cause structural disorder and affect the electronic properties of the material.

### 2.2. Surface Instability

In addition to their inherent instability, PNCs are connected to the ligand capping via ionic interactions. Ligands play a crucial role in stabilizing the PNCs and preventing their agglomeration. However, the connection between the PNCs and ligands is inherently unstable, which can result in the detachment of the ligands from the nanocrystals. In [77], the surface ligands on CsPbBr_3_ nanocrystals were highly dynamic, indicating that the ligands in the perovskite nanocrystal solution may not be tightly bound to the nanocrystal surface. There is a rapid exchange between the free and bound states of the ligands, making them easily lost during separation and purification process.

### 2.3. External Factors

Environmental interactions, including moisture, oxygen, and light, also play significant roles in PNC degradation.

When PNCs are exposed to humid conditions, the color of perovskite turns from black to yellow, and the phase of perovskite grains transitions from the α-phase (cubic phase) to δ-phase (orthorhombic phase), as depicted in Figure 1c [77,78,79]. This transformation occurs due to the introduction of H_2_O into the perovskite through surface leakage or pinholes, where it reacts with MA^+^ and forms weak hydrogen bonds. Consequently, the bonds between the crystal components dissociate, leading to a structural shift of the perovskite from a three-dimensional (3D) system to a zero-dimensional (0D) system [77]. Even in the case of all-inorganic PNCs such as CsPbI_3_, the presence of H_2_O in the air can trigger a transition from the γ-phase to the δ-phase (non-perovskite phase), as shown in Figure 1c [80].

Oxygen in the air is another factor that influences perovskite stability. Perovskite based on Pb shows good stability. However, when the perovskite device is exposed to long-term illumination, such as with CsPbBr_3_ nanocrystals, it shows a color change from green to yellow, which indicates perovskite grain structure decay [78]. Through experiments and calculations, Haque et al. believed that, in perovskite crystals, photostimulation of perovskite leads to the generation of electrons and holes, and, when oxygen diffuses into the grain lattice, it forms a superoxide and reacts with perovskite, causing degradation [79].

Encapsulation and other protection methods can prevent H_2_O and oxygen from entering the air. However, for photovoltaic devices, such as perovskites or photodetectors, light is an inevitable factor that influences perovskite stability during operation. Perovskite nanocrystal devices require long-term stability under continuous illumination for commercial purposes. In a study by McGehee et al., under continuous illumination, the MAPbI_3−x_Br_x_ film X-ray diffusion (XRD) spectrum showed a split perovskite peak, indicating that the perovskite transformed into a different phase. After a few minutes in the dark, the XRD spectrum exhibited a normal complete peak [80].

In addition to light, high temperature is another unavoidable factor that influences the stability of perovskite devices, especially of perovskite solar cells. Owing to the different tolerances of A site ions at high temperatures, metal-ion-based perovskites have better stability than organic ion perovskites. According to thermogravimetry analysis, CH_3_NH_2_ from MAPbI_3_ or MAPbBr_3_ sublimates at 250 and 220 °C, respectively [81,82]. For CsPbX_3_, the decay temperature is approximately 500 °C [81]. In addition, high temperatures accelerate the decay generated by H_2_O and oxygen. Gąsiorowski and colleagues introduced a method for the quantitative and rapid assessment of perovskite layer degradation [83]. They employed optical reflectance measurements in the near-infrared (NIR) range (900–1700 nm) to examine the aging process of perovskite structures over time. Additionally, they monitored alterations in the optical reflectance spectra of newly produced perovskite solar cell structures for a duration of one month.

The observed spectral changes were primarily attributed to the physical mechanism of iodine vapor generation (I), which has low defect formation energies and is a common pathway for degradation [84]. During storage under ambient conditions, iodine vapor may be released from the perovskite lattice, leading to further degradation [85].

## 3. Strategies to Enhance the Stability of PNCs

To address the stability issues of PNCs, researchers have proposed various strategies, including structural modification, defect and impurity control, and encapsulation.

### 3.1. Encapsulation

Encapsulation is the most direct and effective method for improving PNCs and has become a favored optimization technique among researchers in recent years [86]. Since the last century, the use of organic polymers to encapsulate nanomaterials for performance enhancement has gradually become a mainstream technology [86]. Recently, the use of inorganic materials, such as silica dioxide, to encapsulate perovskite nanomaterials has progressively emerged as a promising commercialization-ready technology [87].

**Figure 2:**
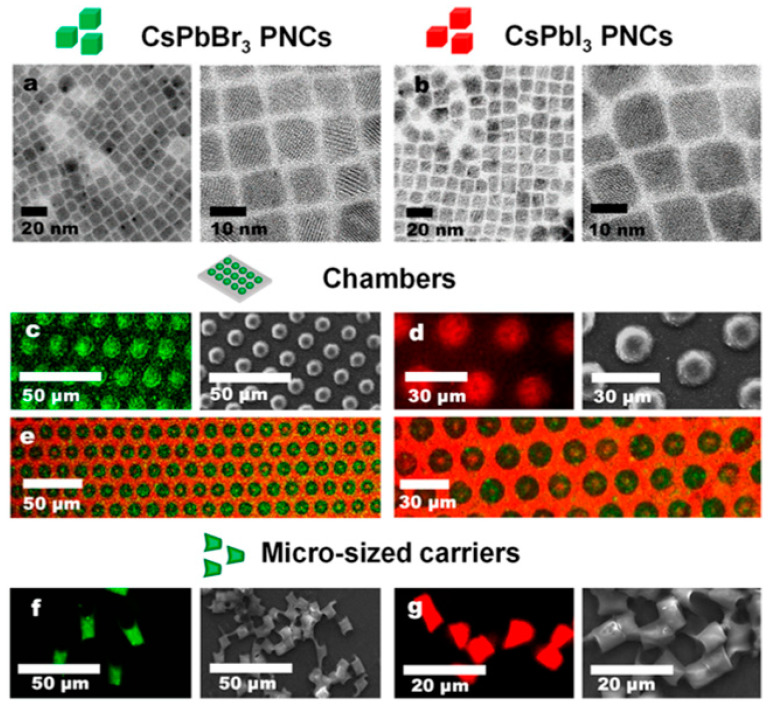

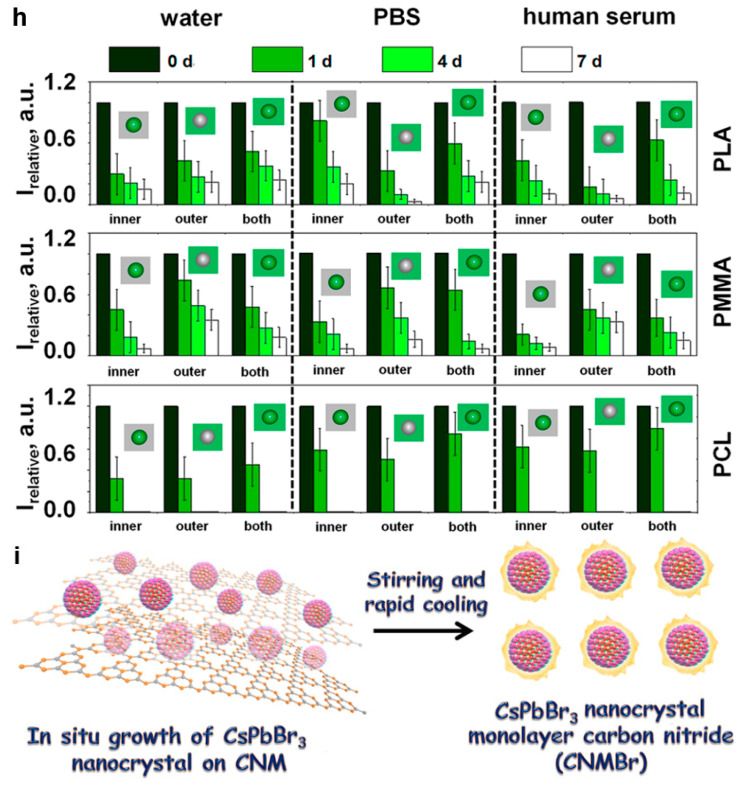
(**a**) Representative transmission electron microscopy (TEM) and high-resolution transmission electron microscopy (HRTEM) images of CsPbBr_3_ PNCs. (**b**) Representative TEM and HRTEM images of CsPbI_3_ PNCs. (**c**) Representative confocal laser scanning microscopy (CLSM) (**left**) and SEM (**right**) images of polylactic acid (PLA) chambers loaded with CsPbBr_3_ PNCs (inner). (**d**) Representative CLSM (**left**) and SEM (**right**) images of PLA chambers loaded with CsPbI_3_ PNCs (inner). (**e**) Representative CLSM images of PLA chambers loaded with CsPbBr_3_/CsPbI_3_ (inner/outer) PNCs. (**f**) Representative CLSM (**left**) and SEM (**right**) images of PLA micro-sized carriers loaded with CsPbBr_3_ PNCs. (**g**) Representative CLSM (**left**) and SEM (**right**) images of PLA micro-sized carriers loaded with CsPbI_3_ PNCs. (**h**) Relative photoluminescence (PL) intensity of CsPbBr_3_ PNCs loaded in PLA/PMMA/PCL chambers (inner, outer, and both) incubated in water, PBS, and HS for varying durations (0, 1, 4, or 7 days). Reproduced from [89] with permission of the American Chemical Society. (**i**) In situ synthesis of carbon nitride protected CsPbBr_3_ (CNMBr) using CsPbBr_3_ and monolayered graphenic carbon nitride (CNM). Reproduced from [91] with permission of Elsevier.

Avugadda et al. proposed a method to achieve well-stabilized PNCs using a low-molecular-weight polymer, polystyrene-block-poly(acrylic acid) (PS-bPAA), as the encapsulating amphiphilic polymer [88]. In the presence of nanocrystals in toluene, the addition of methanol to a single-phase system enables the formation of polymer capsules containing nanocrystals. This approach is suitable for Cs-oleate-coated nanocrystals, which have a smaller steric hindrance and are more conducive to the intercalation of polystyrene (PS). In addition, this method reduces the toxicity of PNCs. The authors assessed the cytotoxic effect of Cs-oleate-coated nanocrystal capsules on an in vitro cell model and found that, after 72 h of material exposure (0.3 μgPb/mL), no significant toxicity was observed in cell viability assays. Talianoy et al. developed a universal method for effectively encapsulating PNCs, CsPbBr_3_, and CsPbI_3_, into well-ordered polymer microchambers and microscale polymer carriers, rendering them water-resistant [89]. Confocal laser scanning microscopy images showed that both the microchambers (Figure 2c–e) and the individual microscale carriers (Figure 2f,g) exhibited strong emissions under continuous-wave laser excitation (488 nm for CsPbBr_3_ or 633 nm for CsPbI_3_), indicating the stability of the PNCs (CsPbBr_3_ and CsPbI_3_) after encapsulation into microchambers or microscale carriers. Different types of PNCs (CsPbBr_3_ and CsPbI_3_) could be loaded together into the same chamber, as shown in Figure 2c–e. The development of organic polymer encapsulation technology indicates that the application of PNCs in biology is becoming more widespread. The relative PL intensity stability measurement incubated in water for 7 days is shown in Figure 2h. Owing to the poor stability of the most commonly used PNCs capped with oleic acid (OA) and oleylamine (OAm) in polar solutions, Chen et al. developed alcohol-stable polyacrylic acid-capped CsPbBr_3_ PNCs (PAA-PNCs). The PNCs were then encapsulated in situ with polystyrene (PS) shells by polymerizing styrene monomers on the PNC surface in a polar organic solvent (e.g., ethanol). The resulting in situ PS-encapsulated PAA-PNCs (i.e., PAAPNCs@iPS) exhibited excellent monodispersity, remarkable water, heat, and UV stability, high fluorescence activity, and color purity [90]. The overall light extraction efficiency from PNCs can be compromised due to light scattering or absorption caused by the encapsulation layer, depending on the selected polymer and encapsulation method. This limitation can impact the performance of optoelectronic devices that rely on light emission or absorption.

Cao et al. employed tetramethoxysilane as a single molecule, which was hydrolyzed in an ammonia solution to produce silica and subsequently formed composites at room temperature. The resulting CsPbBr_3_/SiO_2_ composites consisted of uniformly distributed particles without any noticeable formation of large aggregates [92]. Huangfu et al. first used the fast hydrolysis of (3-aminopropyl)triethoxysilane as a precursor to form a SiO_2_ network for silica dioxide encapsulation. This method was utilized for the in situ growth of nanocrystals and silica on poly (vinylidene fluoride) (PVDF) membranes. After 104 days under ambient conditions and in high-humidity environments with 90% relative humidity, the CsPbBr_3_ nanocrystal PVDF membranes maintained 88.11% of their fluorescence intensity [93]. For hybrid organic–inorganic perovskites, silica encapsulation is an excellent choice for enhancing stability. Azar et al. employed a 3-aminopropyl triethoxysilane (APTES)-assisted reprecipitation and sol–gel method to prepare stable α-FAPbI_3_ nanocrystals at room temperature embedded within a silica matrix [94]. In this case, acetonitrile was used as the solvent for formamidinium iodide instead of dimethylformamide. APTES was used to promote the dissolution of PbI_2_, and, during the crystallization of the perovskite, autocatalytic gelation of APTES was achieved through hydrolysis and condensation to form silica dioxide encapsulation. Compared to previous studies, the authors prepared white LEDs with the highest color gamut (NTSC 144%) reported to date. Although silica encapsulation can bring excellent stability, it can also introduce light scattering, which may reduce light extraction efficiency from nanocrystals. Silica encapsulation adds a rigid layer around the PNCs, which may limit their flexibility and strain tolerance. In applications where flexibility or bending is required, such as flexible electronics or wearable devices, the brittleness of the silica encapsulation can be a disadvantage.

Recently, several novel encapsulation substances and methods have been developed. Gao et al. successfully prepared highly stable CsSnCl_3_ nanocrystals using bone gelatin as a surface capping agent, allowing the PL intensity to be maintained at 95% in water over 55 h [95]. During the growth of the nanocrystals, bone gelatin effectively prevented the oxidation of Sn^2+^ to Sn^4+^, resulting in a PLQY of 3.13% for bone gelatin–CsSnCl_3_ nanocrystals, which was significantly higher than the 2.17% observed for the uncapped counterparts. Furthermore, because of the abundant hydrophilic carboxyl and amino groups in bone gelatin, the coated CsSnCl_3_ nanocrystals exhibited excellent water solubility, indicating that these stable bone gelatin–CsSnCl_3_ nanocrystals hold great potential for applications in the development of fluorescent sensors and detectors. Laishram et al. employed a monolayered graphenic carbon nitride (CNM) material for in situ encapsulation to enhance the stability performance of CsPbBr_3_ nanocrystals [91]. They simulated the Fourier-transform infrared (FTIR) and Raman spectra of carbon nitride using a quantum chemistry package and compared them with experimental vibrational spectra to confirm the presence of the carbon nitride framework in the CNM nanosheets following exfoliation and shell formation, as shown in Figure 2i. Strong spectroscopic evidence showed an increase in crystallinity and a decrease in disorder in the CNM shell. The resulting core–shell CNMBr nanocrystals exhibited stability in air, water, and polar solvents for over 2 months while retaining their fluorescence properties. Wang et al. developed a phase separation strategy to create highly luminescent PNC–polymer composites with a superhydrophobic micro- or nanostructured surface [96]. Ethyl α-cyanoacrylate monomers (ECA) and octavinyloctasilasesquioxane (OVS) were added to a PNC toluene solution. The high reactivity of ECA toward H_2_O led to the rapid anion polymerization reaction in a humid environment, forming the poly(ethyl α-cyanoacrylate) (PECA) polymer matrix as the first phase. As toluene evaporated, the OVS condensed into micro- or nanoscale protrusions on the PECA substrate, creating a second phase and uniformly encapsulating the PNCs within the two polymers. The resulting PNC–PECA–OVS composites showed a significant improvement in chemical stability and increased quantum efficiency. After 1000 h, the PL intensity of the PNCs remained at approximately 60%, whereas the pristine PNCs completely lost their luminescence after only 10 min. Inspired by the protective mechanism of plant leaves, Karabel et al. employed natural carnauba wax as an encapsulation material for nanocrystals to replace the commonly used octadecane [97]. The PNCs prepared using this method exhibited excellent thermal and water stability, and the fabricated nanomaterials could be applied to glass melt printing, allowing for the easy processing of 2D and 3D macrostructures.

Zhang et al. developed a thermal diffusion encapsulation method using ZSM-5 hierarchically structured zeolite (HSZ) as the encapsulation material to optimize the stability of PNCs [98]. The highly water-stable and stable luminescent performance of the encapsulated nanomaterials can be attributed to the interconnected micro/mesoporous network of the ZSM-5 HSZ, where the micropores serve as a shielding wall to isolate the CsPbBr_3_ PQDs from the external environment. The nanocrystals retained 92% of their emission intensity even after being submerged in water for 100 days.

### 3.2. Ligand Engineering

Ligands are organic molecules that bind to the surfaces of PNCs, thereby providing passivation and altering their physicochemical properties [99]. However, PNCs, particularly CsPbBr_3_ nanocrystals, exhibit instability largely because of the interaction between bromide ions and common ligands, such as OAm or protonated OA and its salts (oleate) [100,101]. These interactions result in the dissociation of bromide ions from the nanocrystal surface. Additionally, OAm contributes to the degradation of the PNCs by coordinating with and dissolving lead oleate, causing instability in these materials. This instability leads to a rapid decrease in PLQY and the formation of polycrystalline aggregates [100].

Ligand engineering is a promising strategy to enhance the stability of PNCs by modifying their surface chemistry. This approach involves the use of organic ligands or molecules to passivate the surface of the nanocrystals, thereby improving their physicochemical properties, including stability, solubility, and PL intensity [102,103,104,105,106,107,108]. In ligand engineering, various organic molecules with functional groups, such as carboxylic acids [109,110], amines [110,111,112], phosphonic acids [113,114], and thiols [115], have been used to bind the surface atoms of PNCs. This passivation helps prevent degradation caused by external factors such as moisture, oxygen, temperature, and light exposure. Additionally, modified ligands can improve the dispersibility of the nanocrystals in various solvents, enabling their use in a wide range of applications [102].

**Figure 3:**
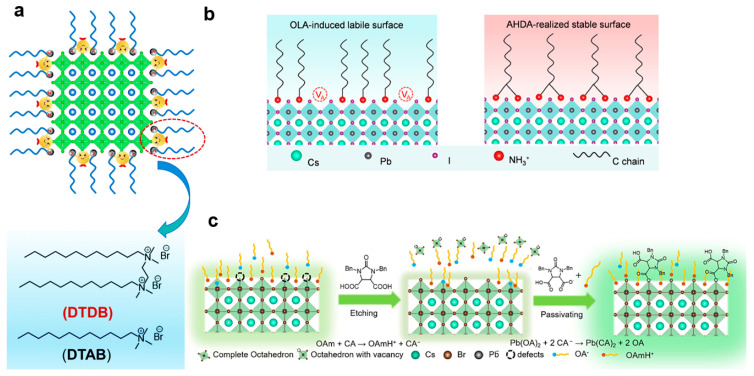
(**a**) Schematic diagrams of the interaction between the CsPbBr_3_ nanocrystals and the DTDB ligand, and the chemical structure of the DTDB and DTAB ligands. Reproduced from [118] with permission of the Royal Society of Chemistry. (**b**) Ligand content (wt.%) at the surface of OLA- and AHDA-PNCs determined from TGA curves after several purification cycles. Reproduced from [121] with permission of the American Chemical Society. (**c**) Schematic representation of the effect of CA ligand enhancement and recovery of PL intensity and stability by multifunctional etching ligand treatment for PNCs. Reproduced from [122] with permission of Elsevier.

The LARP technique under ambient conditions was identified as a simple and scalable method for synthesizing lead halide PNCs [41,116,117]. Li et al. employed a room-temperature LARP method to synthesize CsPbBr_3_ nanocrystals using N1,N2-didodecyl-N1,N1,N2,N2-tetramethylethane-1,2-diaminium bromide (DTDB) as both a ligand and an additional bromine source [118]. The ligand could be tightly anchored onto the nanocrystal surface through dual N^+^-binding sites, as shown in Figure 3a. The synthesized DTDB–CsPbBr_3_ nanocrystals exhibited a high PLQY of 92.3%, maintained excellent colloidal stability under stringent purification conditions, and could be reused up to 10 times. Similarly, by employing the LARP method, Cai et al. used cetyltrimethylammonium bromide as a ligand instead of the original OA and OAm, resulting in more stable PNCs with a PLQY of 77% [119]. Following their work using the LARP method, Cai et al. reported a new X-type ligand, organic sulfonium bromide [120]. Three representative ligands (DAM, DSM, and DMM) were obtained by reacting dodecylmethylsulfide with allyl bromide, (3-bromopropyl)trimethoxysilane, and 1,4-dibromobutane, respectively. Subsequently, they prepared CsPbBr_3_ PNCs using the LARP method, in which DSM and DMM endowed the CsPbBr_3_ PNCs with enhanced thermal and light stabilities stemming from the cross-linkable or bidentate ligand structure [120].

Recently, there has been a continuous emergence of quite innovative ligands and synthesis methods. Pan et al. introduced organic semiconductor molecules into CsPbBr_3_ nanocrystals, resulting in CsPbBr_3_ nanocrystals with a high photoluminescence quantum efficiency of 82% [123]. Furthermore, these nanocrystals survived commercial accelerated operation stability tests, such as high temperature (85 °C) and high humidity (85%), and they maintained over 80% of their initial PL intensity for more than 1000 h. Note that these semiconductor molecules could form type II heterostructures with CsPbBr_3_ nanocrystals, protecting them from damage caused by photoexcited carriers [123]. Zeng et al. utilized a polyamine chelating ligand, N′-(2aminoethyl)-N′-hexadecylethane-1,2-diamine (AHDA), to achieve highly stable colloidal CsPbI_3_ PNCs [121]. The scheme is shown in Figure 3b. The protonated AHDA can stabilize the PNC surface lattice with a high binding energy of 2.36 eV, significantly higher than the 1.47 eV achieved by commonly used oleoyl amide ligands. Chelation significantly suppressed the dynamic detachment of surface ligands, enabling the CsPbI_3_ PNCs to remain stable under various environmental stimuli [121]. Additionally, according to the authors’ experiments, this ligand can be employed for the synthesis of other PNCs, such as CsPbBr_3_ and FAPbI_3_.

Shu et al. proposed an effective ligand engineering strategy using octylamine-modified polyacrylic acid (OPA) as a coating ligand for CsPbBr_3_ nanocrystals, prepared through a modified ligand-assisted precipitation method [124]. The strong binding affinity between OPA, oleic amines, and CsPbBr_3_ nanocrystals reduced the surface trap states, and the abundance of carboxyl groups in OPA imparted good water solubility to the nanocrystals [124]. Moreover, OPA can control the particle size of nanocrystals. The improved PNCs exhibited significantly enhanced water stability, photostability, and thermal stability, with the fluorescence maintaining 80.13% of its original value after 15 days of immersion in water [124]. Min et al. proposed a strategy for surface modification of CsPbI_3_ nanocrystals using a long-chain ligand with a thiol group, 1-octadecanethiol (ODT) [125]. The presence of a thiol group in the ODT ligand strengthened the coordination interaction between the ligand and nanocrystals. The crystal phase of the CsPbI_3_ nanocrystals remained unchanged after the ODT treatment, and density functional theory calculations showed that the binding energy was maximized when one ODT ligand and one OA ligand were bound to the Pb^2+^ ions [125]. The ODT-treated nanocrystals achieved a PLQY close to 100% and demonstrated excellent resistance to continuous ultraviolet light exposure [125]. Zhao introduced 1,3-bisbenzyl-2-oxoimidazolidine-4,5-dicarboxylic acid (cyclic acid, CA) as an etchant and ligand [122]. The structure of the CA is shown in Figure 3c. As a soft X-type ligand, CA can form multisite bonds with the surface of the PNCs, whereas the benzylic branched chains can regulate the morphology of the PNCs. By removing incomplete octahedra [PbX_6_]^4−^ and simultaneously passivating surface defects, the PLQY of the PNCs increased from 76% to 95%. The stability of the PNCs in polar solvents, humidity, heat, and light was enhanced [122]. Jin et al. proposed a strategy involving the use of an amphoteric ionic polymer (PCA) to treat perovskite quantum dots in polar green solvents [126]. Compared to OA molecules, experimental and theoretical results showed that PCA molecules had stronger coordinating interactions with the surface of perovskite quantum dots, leading to more effective passivation of their defect electrons [126].

Liu et al. reported a bis(trifluoromethane)sulfonimide superacid molecule that enhanced the PL intensity of metal halide PNCs [127]. After superacid treatment, the photoluminescence quantum efficiency of the CsPbI_3_ nanocrystals increased significantly from 28.6% to nearly 100%. The improved PLQY of the CsPbX_3_ nanocrystals mainly originated from surface passivation. The CsPbX_3_ nanocrystals were subsequently modified with PMMA, which could greatly improve their stability while maintaining high PL intensity and good dispersion [127].

Inorganic ligands also perform well in the modification of PNCs and the passivation of defects. Zang et al. employed tungstosilicic acid for surface modification of cesium lead halide nanocrystals [128]. In this process, TSA binds the hydrophobic chains of OAm to the surface of CsPbBr_3_ nanocrystals while altering the charge distribution of the cationic surface layer, thereby enhancing the interaction with surface traps [128]. TSA-modified CsPbBr_3_ nanocrystals demonstrated long-term environmental stability and excellent tolerance to polar solvents. After storage at room temperature for 50 days, approximately 80% of the initial luminescence intensity was maintained [128].

Ligand engineering is an essential optimization method for synthesizing unconventional PNCs. For Cs_2_Ag_1−x_NaxInCl_6_ double perovskite (DP) nanocrystals, Ahmad et al. used a strongly coordinating silver–trioctylphosphine complex and additional TOP ligands, allowing the synthesis of high PLQY and stable DP nanocrystals [129]. The Ag–TOP complex acted as a highly reactive silver precursor, preventing silver ions from reducing to metallic silver. During the nucleation and growth stages, TOP promoted nucleophilic reactions with benzoyl chloride, forming a benzoyl tris(octyl)phosphonium chloride intermediate, which served as both a halide source and surface-capping ligand, enabling the formation of high-quality DP nanocrystals [129]. DP nanocrystals exhibited high tolerance to common antisolvents such as methyl formate and isopropanol, which was attributed to the tight binding of the dicationic ligand benzoyl tris(octyl)phosphonium and oleate ammonium cations and oleate anions on the surface of DP nanocrystals [129].

Ligand engineering, despite its benefits, is not without flaws. It often entails intricate synthetic procedures to achieve desired ligand modifications. While it can enhance the stability of PNCs, it can also present new challenges. Careful consideration must be given to the choice of ligands and their stability under operating conditions to prevent degradation or decomposition issues. Moreover, ligand engineering can introduce variability in the properties of PNCs due to the sensitivity of the ligand modification processes. In certain conditions, achieving reproducible results across different batches or laboratories can be challenging, necessitating meticulous control over synthesis and characterization parameters.

### 3.3. Metal Cation Dopants

**Figure 4:**
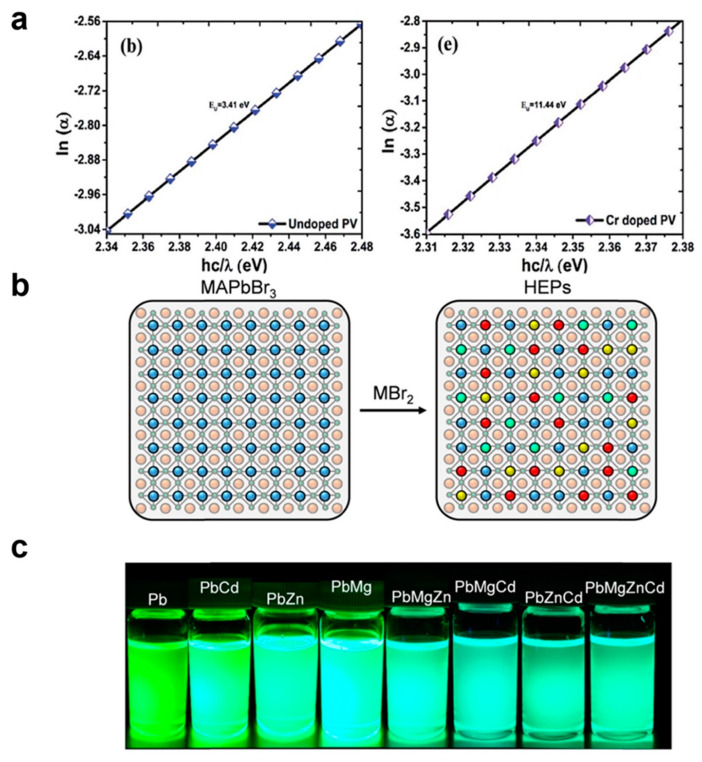
(**a**) Urbach energy plot of pristine and Cr-doped PNCs. Reproduced from [138] with permission of the Royal Society of Chemistry. (**b**) Schematic diagram showing a possible reaction that occurs when three additional elements are doped in the perovskite lattice upon mixing with a metal halide powder blend. (**c**) Representative photographs of synthesized colloidal solutions under UV excitation. Each sample is labeled with the B site elements, e.g., PbZnCd for MA(PbZnCd)Br_3_ HEP nanocrystals. Reproduced from [140] with permission of the American Chemical Society.

Metal cation doping is a common approach for enhancing the stability of PNCs [130,131,132,133,134,135,136]. Mohapatra et al. reported the synthesis of highly luminescent green-emitting zinc-doped cesium lead titanate nanocrystals under high-humidity conditions (RH > 60%) using the LARP method [137]. Red-emitting zinc-doped CsPbBr_x_I_3−x_ nanocrystals were synthesized from preprepared zinc-doped CsPbBr_3_ nanocrystals through a halide exchange process. The incorporation of zinc effectively adjusted the bandgap, passivated Pb^2+^ defects, and enhanced the stability of the nanomaterial [137]. In Nazim’s study, Cr^3+^ cations were used as dopants in the growth of metal-doped hybrid organic–inorganic metal halide PNCs [138]. The study found that proper doping with small Cr^3+^ cations could partially replace Pb^2+^ ions in the PNCs, enhancing their optical properties (estimated by Urbach plots, as shown in Figure 4a) while maintaining their cubic geometry and high thermal stability. Specifically, after metal ion doping, the PNCs were able to suppress the surface grain boundaries and recombination losses through crystal defect control [138]. Consequently, Cr^3+^ ion doping may result in novel doped organic metal halide perovskite rod-shaped nanocrystals with slightly altered lattice parameters and a retained cubic phase [138]. Deng et al. developed a simple low-temperature method to synthesize CsPb_2_Br_5_/CsPbBr_3_ PNCs doped with different transition metal (II) ions (Ni^2+^, Cu^2+^, and Zn^2+^) [139]. This metal site doping strategy effectively eliminates bromine vacancies and suppresses nonradiative recombination. As a result, these transition metal (II)-doped CsPb_2_Br_5_/CsPbBr_3_ perovskite nanocrystals exhibited a higher PLQY than undoped nanocrystals [139]. Films based on Ni^2+^-doped nanocrystals demonstrated significant water stability (60 days), whereas Cu^2+^-doped nanocrystals exhibited outstanding thermal stability (up to 150 °C). The anionic exchange of these transition metal (II)-doped CsPb_2_Br_5_/CsPbBr_3_ nanocrystals resulted in high luminescence across the entire visible spectrum [139].

Solari et al. proposed a post-synthesis approach to stabilize lead-reduced metal halide PNCs through high-entropy alloying [140]. As shown in Figure 4b,c, this method enables easy access to the compositional space without concerns about the solubility constraints of the metal precursors in the anti-solvent, which can be cumbersome for many hot injection and post-synthesis techniques. This is particularly suitable for investigating high-entropy perovskites and their synthesis. By doping the nanocrystals with multiple elements (Cd^2+^, Zn^2+^, and Mg^2+^) at relatively high concentrations, the resulting high-entropy perovskite nanocrystals exhibited good colloidal stability and narrow-band emission, along with even higher PLQY (η_PL)_ and shorter fluorescence lifetimes (τ_PL)_ [140]. In the high-entropy alloying synthesis, the formation of intermediate states and doped phases stabilizes the lead-reduced perovskite lattice. Li et al. synthesized Al^3+^-doped Cs_2_AgBiCl_6_ [141]. The incorporation of Al^3+^ ions facilitated the transition from an indirect to a direct bandgap, resulting in enhanced fluorescence in the nanocrystals [141]. Furthermore, Al^3+^-doped Cs_2_AgBiCl_6_ demonstrated exceptional stability, maintaining its original structure for over 7 months.

Table 1 shows the stability performance of different metal ion doping. It can be seen that, although metal ion doping can effectively enhance the thermal stability and stability in humid environments of nanoparticles, in line with previous content, it is undeniable that it has significant limitations. Certain metal cations may not be universally applicable to different PNCs.

### 3.4. Optimization of the Fabrication Process

In the process of synthesizing PNCs, researchers have not only focused on the selection of materials but also explored various synthesis methods and treatments. For example, precursor optimization [142,143,144], post-treatment [145,146,147], metal–organic frameworks [148,149,150,151], and in situ growth [152,153,154,155] are some of the strategies employed.

In Syed’s research, the authors developed a simple, completely amine-free colloidal synthesis method using a hot injection approach [156]. They introduced octyl bromide as a bromine source under open atmospheric conditions to prevent the formation of unstable oleoyl amine bromide and to avoid amine and ammonium species in the reaction medium. By varying the Pb/Br ratio, it was observed that an excess or high amount of halogens (bromide ions) could suppress the nonradiative recombination process and contribute to the subsequent surface passivation of the CsPbBr_3_ PNCs. A molar ratio of Cs/Pb/Br = 1:6:24 was suitable for producing highly stable CsPbBr_3_ PNCs, which maintained 66% of their initial PL intensity even after 5 months under ambient conditions [156]. Sumanta et al. demonstrated that, by using NH_4_I as an additional precursor in the conventional hot injection method, they could obtain phase-pure, monodisperse, stable, and highly luminescent CsPbI_3_ nanocrystals [157]. The outstanding characteristics of these CsPbI_3_ nanocrystals were found to be a result of NH^4+^ replacing some Cs^+^ ions on the iodine-rich surface of the nanocrystals, serving as both a halide precursor and a surface capping agent [157]. In Sanat’s study, a low-cost and highly reactive bromide precursor, 1,3-dibromo-5,5-dimethylhydantoin (DBDMH), was chosen as the bromide precursor [158]. In their synthesis approach, they employed a three-precursor hot injection method, where DBDMH was used as the bromide precursor. DBDMH reacted with OA to release HBr, which subsequently reacted with oleamide to form oleoylammonium bromide (OAmBr) [158]. In this reaction, OAmBr served as the active bromine source [158].

**Figure 5:**
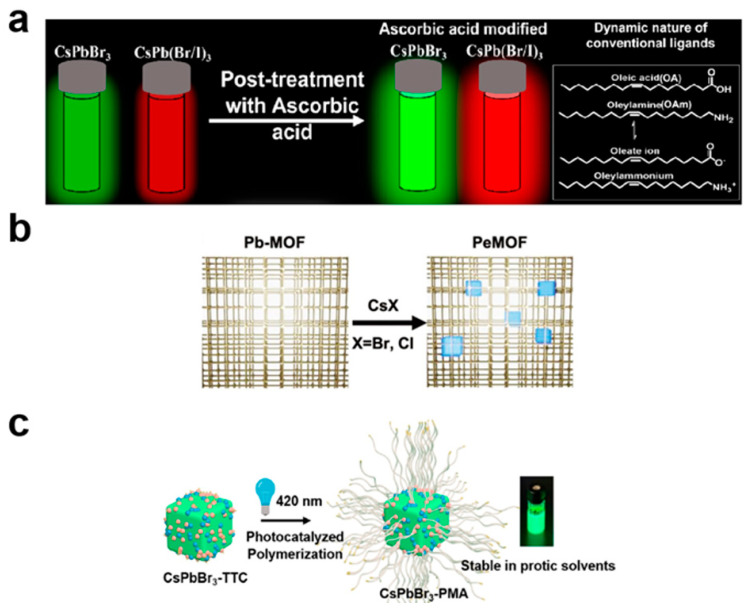
(**a**) Schematic representation of surface treatment of CsPbX_3_ PNCs with ascorbic acid. Reproduced from [161] with permission of the American Chemical Society. (**b**) Schematic illustration of the perovskite metal–organic framework (PeMOF) fabrication process. Reproduced from [163] with permission of Wiley. (**c**) Synthesis schematic core–shell structured CsPbBr_3_–polymer nanoparticles. Reproduced from [164] with permission of the American Chemical Society.

Drawing inspiration from previous studies involving ascorbic acid in the synthesis of copper nanoparticles [159,160], Mishra’s group achieved high-quality CsPbX_3_ nanocrystals through a simple and accessible post-treatment method using ascorbic acid [161]. The scheme of this post-treatment process is shown in Figure 5a. Ascorbic acid surface treatment significantly improved the PL intensity and stability of the CsPbBr_3_, CsPb(Br/I)_3_, and CsPbI_3_ nanocrystals. By analyzing the photoluminescence decay curves of the nanocrystals, researchers discovered that effective surface passivation by ascorbic acid was due to the presence of Pb–O interactions. These interactions limited the activity of oleamine, resulting in improved stability of the ascorbic acid-treated nanocrystals at various temperatures [161]. Mishra’ group conducted a convenient post-treatment of amine-free CsPbBr_3_ PNCs using PbBr_2_ [162]. The positive effects of the PbBr_2_ treatment were manifested in filling the Pb^2+^ and Br^-^ vacancies on the PNC surface, thereby significantly improving the PL intensity and lifetime. The PbBr_2_-treated amine-free CsPbBr_3_ PNCs demonstrated significant improvements, retaining 66% of their initial PL even after 6 h of water treatment.

Tsai et al. introduced a novel method for preparing CsPbX_3_ nanocrystal films for use in stable blue LEDs [163]. By incorporating CsX (X = Br or Cl) into metal–organic framework (MOF) films containing Pb metal nodes (Pb–MOF), 10–20 nm CsPbX_3_ nanocrystals could be formed, which were surrounded by the MOF matrix, as shown in Figure 5b. Compared with CsPbBr_3−x_Cl_x_ films, the luminescence of Cs–PeMOF could withstand higher laser power irradiation and resist photoinduced ion migration [163]. Time-of-flight secondary ion mass spectrometry (ToF-SIMS) depth profiling revealed a uniform compositional distribution inside the Cs–PeMOF film under high voltage and injection current stress [165], confirming the limitations of the ion migration kinetics within the PeMOF nanocomposite, thereby achieving outstanding operational stability [163].

Jin et al. introduced a method for the controlled free-radical photocatalytic polymer brush growth on the surface of CsPbBr_3_ PNCs using a grafting approach, as shown in Figure 5c [164]. In this process, the nanocrystals act as both a photocatalyst and an initiator-bound substrate for synthesizing polymers with well-defined molecular weights and low dispersity [164]. The formed core–shell structure of the CsPbBr_3_–polymer nanoparticles exhibited improved colloidal and optical stability of the CsPbBr_3_ core in various polar organic solvents, in water, and under ultraviolet radiation condition [164]. Chen et al. reported a simple strategy to prepare tunable green-emitting (517–528 nm) CsPbBr_3_ perovskite nanocrystal glass by employing an in-situ growth method with fine control of the glass precursor concentration and heat treatment temperature [166]. The precursor glass was prepared using the melt-quenching method. In situ crystallization within the glass matrix resulted in green-emitting CsPbBr_3_ perovskite nano glass (PNG) and red-emitting CsPbBr_x_I_3−x_ PNG with continuously adjustable emission wavelengths [166].

In addition to traditional optimization methods, optimization synthesis approaches have been increasingly accepted in recent years. Electrospinning is a popular synthetic technique. Tabassum et al. prepared poly(l-lactic acid) (PLLA) nanofiber membranes embedded with FAPbBr_3_ PNCs using electrospinning [167]. This is a simple and low-cost technique for directly confining nanoscale functional materials within continuous polymer nanofibers. PLLA, as a polymer matrix, provides a high-surface area framework for completely encapsulating perovskite nanocrystals [167]. Furthermore, FAPbBr_3_ nanocrystals spontaneously crystallized within the PLLA nanofibers. The resulting PLLA–FAPbBr_3_ nanofiber membranes were stable and maintained in water for approximately 45 days without significant decomposition [167]. In a study by Liang et al., the authors utilized electrospinning guidance to develop a one-step strategy for the continuous production of stable chiral PNCs with a high PLQY and circularly polarized luminescence [168]. The electrospinning process employed polyacrylonitrile (PAN) nanofiber spinning as a reactor, generating in situ tailored chiral MAPbBr_3_ PNCs using R-(+)-methylbenzylammonium bromide (R-MBABr) or S-(−)-methylbenzylammonium bromide (S-MBABr). The resulting R(S)-MBABr-modified MAPbBr_3_ PNCs or PAN nanofiber membranes, referred to as R(S)-PNCs or PAN nanofiber membranes, exhibited up to 88% green luminescence and maintained 60% of their PLQY after 180 days of storage in the atmosphere [168]. Khurana et al. demonstrated a route to design CsPbBr_3_–PbSe nano-heterostructures and other perovskite nano-heterostructure derivatives using defect-rich MoSe_2_ nanosheets, as well as investigated the influence of PbSe nanoparticle size on their optical properties [169]. In this synthesis route, PbSe nanoparticles were formed in the early stages of the reaction through a unique cation-exchange process, with CsPbBr_3_ nanocrystals epitaxially growing on top of them. Using this method, a nearly threefold enhancement in PL intensity was achieved, and the resulting CsPbBr_3_–PbSe NHSs exhibited improved stability in the presence of water and remained stable for several months at room temperature without decomposition [169].

Additionally, various synthesis optimization methods can be combined. In Xu’s study, the authors effectively suppressed photoinduced phase separation in mixed halide nanocrystals through a synergistic approach of precursor and surface engineering [170]. High-quality mixed halide perovskite CsPbBr_3−x_I_x_ nanocrystals were successfully synthesized using DMAPbX_3_ (DMA = dimethylammonium, (CH_3_)_2_NH^2+^) as a precursor through a typical hot injection method. Furthermore, n-octylamine was used to passivate the nanocrystals and further enhance their performance. The resulting nanocrystals exhibited 92% photoluminescence quantum efficiency, high dispersity, and good phase stability under ambient conditions [170]. In Mahato’s study, the authors utilized 5% DMSO as a dopant to synthesize α-CsPbI_3_ in an outdoor setting. They employed spin coating to prepare a perovskite solar cell with a hole-transporting layer based on PEDOT:PSS. This configuration resulted in a maximum power conversion efficiency of 10.6% [171].

## 4. Conclusions

PNCs have enormous potential for application in photovoltaics, photocatalysis, and LEDs. This study reviewed the key factors influencing the environmental and thermal stability of PNCs, as well as the internal and external factors causing PNCs degradation, summarizing the mainstream stability optimization methods in recent years. When it comes to characterizing stability, there are variations among authors in terms of their approaches, methods, and applications in different contexts and articles. Consequently, the data used to assess stability differ, with some relying on PLQY and others using PL intensity. Nonetheless, various authors have managed to enhance the stability of the materials through diverse methods, depending on their respective research.

In practical research, researchers employ various methods to enhance stability in response to different situations. However, in recent years, ligand engineering and encapsulation optimization have emerged as the most popular techniques for improving the stability of PNCs. This is primarily because ligands and encapsulation materials can be modified to protect a wide range of PNCs. Thus, the development of new ligands and materials to protect PNCs is desirable for improving their stability.

Ligand engineering addresses the issue of loose binding between OA or OAm and the surface of PNCs, leading to ligand dissociation and precipitation of Br and other halogens, causing the self-degradation of PNCs. Encapsulation optimization takes advantage of the water and oxygen barrier properties of encapsulation materials to protect the performance of PNCs while addressing external environmental factors, such as humid air causing A site elements or reactions with water and oxygen, preventing PNCs degradation from external causes. Metal cation doping and synthesis optimization focus on the structural characteristics of PNCs, optimizing the perovskite lattice itself, reducing defects, and enhancing PNCs stability while improving their performance, particularly reducing the likelihood of self-degradation. Currently, new technologies to enhance the stability of PNCs are urgently required, and several researchers are continuously improving perovskite nanomaterials. We believe that PNCs will have great commercial value in the future.

## Figures and Tables

**Table 1:** Diverse metal ions as dopants and their impact on the performance stability of perovskite nanoparticles.

Perovskite	Dopant Metal Ion	Performance Stability
CsPbBr_3_	Zn^2+^ [137]	Maintained 91% normalized PL intensity after 60 min heating
CsPbBr_3_	Ni^2+^ [139]	Maintained 71.93% normalized PLQY after 60 days under water
MAPbBr_3_	Zn^2+^, Mn^2+^ [140]	Maintained 92% normalized PL intensity after 30 days
Cs_2_AgBiCl_6_	Al^3+^ [141]	Maintained 92% normalized PLQY after 240 days

## Data Availability

In this review manuscript, no new data were created.
